# Amelioration of Behavioral Abnormalities in BH_4_-deficient Mice by Dietary Supplementation of Tyrosine

**DOI:** 10.1371/journal.pone.0060803

**Published:** 2013-04-05

**Authors:** Sang Su Kwak, Mikyoung Jeong, Ji Hye Choi, Daesoo Kim, Hyesun Min, Yoosik Yoon, Onyou Hwang, Gary G. Meadows, Cheol O. Joe

**Affiliations:** 1 Department of Biological Sciences, Korea Advanced Institute of Science and Technology, Daejeon, South Korea; 2 Department of Food and Nutrition, HanNam University, Daejeon, South Korea; 3 College of Medicine, Chung-Ang University, Seoul, South Korea; 4 Department of Biochemistry and Molecular Biology, College of Medicine, University of Ulsan, Seoul, South Korea; 5 Department of Pharmaceutical Sciences, College of Pharmacy, Washington State University, Pullman, Washington, United States of America; University of Chicago, United States of America

## Abstract

This study reports an amelioration of abnormal motor behaviors in tetrahydrobiopterin (BH_4_)-deficient *Spr* ^−/−^ mice by the dietary supplementation of tyrosine. Since BH_4_ is an essential cofactor for the conversion of phenylalanine into tyrosine as well as the synthesis of dopamine neurotransmitter within the central nervous system, the levels of tyrosine and dopamine were severely reduced in brains of BH_4_-deficient *Spr* ^−/−^ mice. We found that *Spr* ^−/−^ mice display variable ‘open-field’ behaviors, impaired motor functions on the ‘rotating rod’, and dystonic ‘hind-limb clasping’. In this study, we report that these aberrant motor deficits displayed by *Spr* ^−/−^ mice were ameliorated by the therapeutic tyrosine diet for 10 days. This study also suggests that dopamine deficiency in brains of *Spr* ^−/−^ mice may not be the biological feature of aberrant motor behaviors associated with BH_4_ deficiency. Brain levels of dopamine (DA) and its metabolites in *Spr* ^−/−^ mice were not substantially increased by the dietary tyrosine therapy. However, we found that mTORC1 activity severely suppressed in brains of *Spr* ^−/−^ mice fed a normal diet was restored 10 days after feeding the mice the tyrosine diet. The present study proposes that brain mTORC1 signaling pathway is one of the potential targets in understanding abnormal motor behaviors associated with BH_4_-deficiency.

## Introduction

Tetrahydrobiopterin (BH_4_) has been established as an obligatory cofactor for aromatic amino acid hydroxylases including phenylalanine hydroxylases (PAH), tyrosine hydroxylase (TH), tryptophan hydroxylase (TPH), and all isoforms of nitric oxide synthase (NOS) [1]. Since TH and TPH metabolize tyrosine and tryptophan to produce dopamine and serotonin neurotransmitters within the central nervous system, BH_4_ deficiency has often been implicated to be associated with many neurological disorders such as Parkinson’s disease, autism, depression, Alzheimer’s disease, and phenylketonurea [1]–[3].

Studies have suggested that there is interplay between the dysfunction of dopaminergic neurons and behavioral abnormalities in BH_4_-deficient mice. Yang et al. [4] reported severely impaired locomotive activities along with dramatically reduced levels of dopamine in brains of BH_4_-deficient *Spr* ^−/−^ mice. More recently, a correlation among the suppression of dopaminergic development, subsequent deficiency in brain dopamine levels, and motor dysfunction was evidenced in BH_4_-deficient *Spr* ^−/−^ mice [5]. Many behavioral disorders are associated with abnormal neurotransmitter activities. A typical example of these disorders is Parkinson’s disease, a progressive neurodegenerative disease, which is characterized by a deficit in the neurotransmitter dopamine (DA) [6]. The pathological hallmarks of Parkinson’s disease include the loss of dopaminergic neurons in the substantia nigra pars compacta (SNpc) and corresponding decrease in the amount of dopamine production in the striatum (STR), which plays a pivotal role in normal motor function [7]. Major symptoms of Parkinson’s disease thus include tremors in hands, arms, feet, and face, slowness of movement (bradykinesia or akinesia), and difficulties in balance and coordination (rigidity) [8], [9].

Previously, we have demonstrated that BH_4_ deficiency results in the inactivation of mTORC1 pathway in brains of BH_4_-deficient *Spr* ^−/−^ mice due to paucity of a specific amino acid, tyrosine [10]. An evolutionarily conserved protein kinase complex, mTORC1, regulates cell proliferation, cell size, and cell cycle [11], [12]. Activation of mTORC1 pathway stimulates protein synthesis as well as ribosomal biosynthesis, but inactivates catabolic processes such as autophagy [13]–[15]. Recent advances in the study of mTORC1 signaling delineate a potential link between neurological disorders and impaired mTORC1 signaling in the brain. For example, mTORC1 signaling is known to be modified in some pathological states of the nervous system including tuberous sclerosis, cortical dysplasia and neurodegenerative disorders [16]–[18].

Herein, we first report the effects of dietary supplementation of a specific amino acid on the improvement of abnormal motor functions in BH_4_-deficient mice generated by the knockout of the gene encoding sepiapterin reductase (SR), which catalyzes BH_4_ synthesis.

## Materials and Methods

### Experimental Mice and the Dietary Tyrosine Supplementation

*Spr* ^−/−^ mice on a mixed C57BL6/sv129 hybrid background were generated as described elsewhere [4]. Both *Spr* ^+/+^ and *Spr* ^−/−^ mice from the same mother were weaned at 20 days after birth and fed a normal diet *ad libitum* for 5 days to acclimate mice to normal mouse food. For the dietary tyrosine therapy, *Spr* ^−/−^ mice were pretreated with ‘high dose of BH_4_’ (122 µg/g body weight/day), L-DOPA (13.5 µg/g body weight/day), 5-hydroxytryptophan (9.5 µg/g body weight/day), carbidopa (2.5 µg/g body weight/day), ascorbic acid (100 µg/g body weight/day), and N-acetyl-L-cysteine (50 µg/g body weight/day) by oral administration as previously adopted by Elzaouk et al. [19]. After the acclimatization period, *Spr* ^+/+^ or *Spr* ^−/−^ mice 25 days of age were fed either a normal or the therapeutic tyrosine diet in which 5.6% tyrosine (w/w) was added to the normal diet for 10 days. This dietary trial was based on the study by Matalon et al. [20] who has found that a dietary formula enriched in tyrosine is effective in reducing blood phenylalanine concentration in mice. The effects of the dietary tyrosine therapy on motor behaviors, brain tyrosine levels, and dopamine in 35-d-old experimental mice were then examined. All animal experiments were approved by Institutional Animal Care and Use Committee (IACUC) at Korea Advanced Institute of Science and Technology (KA2010-01). For leucine supplementation, 25-d-old mice pretreated with ‘high-dose of BH_4_’ solution were fed the leucine-enriched diet in which 3.6% leucine (w/w) was added to a normal diet for 10 days under *ad libitum* conditions. To explore for the effects of dopamine on motor behaviors, TH protein expression and brain mTORC1 activity, 25-d-old mice were treated with dopamine precursor, L-DOPA (13.5 µg/g body weight/day) was coadministered with carbidopa (2.5 µg/g body weight/day) for 10 days to inhibit peripheral aromatic-L-amino acid decarboxylase (AADC), an important enzyme that metabolizes L-DOPA into dopamine [21].

### Determination of Phenylalanine and Tyrosine Levels in Brains of Experimental Mice

Concentrations of free phenylalanine and tyrosine in the cerebra separated from brains of *Spr* ^+/+^ or *Spr* ^−/−^ mice fed either a normal or the tyrosine diet were measured. Levels of each amino acids were determined by reversed phase high-performance liquid chromatograpy (RP-HPLC) using the Pico-tag method. Phenylisothiocyanate-derived free amino acids were analyzed on a Pico-tag amino acid analysis column with monitoring of the elutes at 254 nm.

### Levels of Brain Dopamine and its Metabolites

Samples of caudate putamen separated from brains of 35-d-old *Spr* ^+/+^ or *Spr* ^−/−^ mice fed either a normal or the tyrosine diet, were homogenized in 0.1 M perchloric acid and the acid soluble fraction was collected. Quantification of striatal dopamine (DA), 3,4-dihydroxyphenylacetic acid (DOPAC), and homovanillic acid (HVA) were measured by HPLC using C18 column (Novapack Coorporation) and detected electrochemically [22].

### Behavioral Analyses

#### Open-field test

Mice were gently placed at the center of the open-field test arena (40×40×40 cm) in a dark room and allowed to explore for a period of 30 min or 1 h. Motor activities were monitored at 5 min intervals in a 1 h time period by using digital video recording. Etho Vision (Noldus) was employed to analyze the video data [23].

#### Rotating rod test

Coordination of motor behaviors were monitored by the rotating rod performance [24]. Mice were placed onto a rotating rod (4 cm in diameter: manufactured by Ugo Basile Biological Research Apparatus, model 47600) that started at 4 rpm and incrementally accelerated at each 20 sec to a final 40 rpm and the latency to fall was recorded. Each experimental mouse performed nine trials over a span of three days (3 trials/day with an interval of 1 h).

#### Hind-limb clasping test

Experimental mice were picked up and suspended by the tail for 25 sec while being videotaped. The duration of hind-limb clasping near the body was measured for each mouse [5].

### Western Blotting

Cerebral or midbrain proteins obtained from 35-d-old experimental mice were separated by 10% SDS-PAGE, blotted onto a nitrocellulose membrane, blocked with 5% BSA, incubated with anti-phospho S6K antibody (Thr389, #9234, 1∶500, Cell Signaling Technology), anti-S6K antibody (SC-8414, 1∶1,000, Santa Cruz Biotechnology), anti-phospho S6 antibody (Ser235/236, #2211, 1∶1,000, Cell Signaling Technology), anti-S6 antibody (#2317, 1∶500, Cell Signaling Technology), anti-TH antibody (1∶3,000, Millipore) or anti-actin antibody (SC1616, 1∶1,000, Santa Cruz Biotechnology), followed by an additional incubation with horseradish peroxide-conjugated anti-rabbit-IgG or anti-mouse-IgG antibodies (Jackson Laboratory). Immunoreactivity was visualized using horseradish peroxide substrate (Dae Myung Science).

## Results

### Recovery of Tyrosine Concentrations in Brains of *Spr* ^−/−^ Mice by the Tyrosine Diet

**Figure 1:**
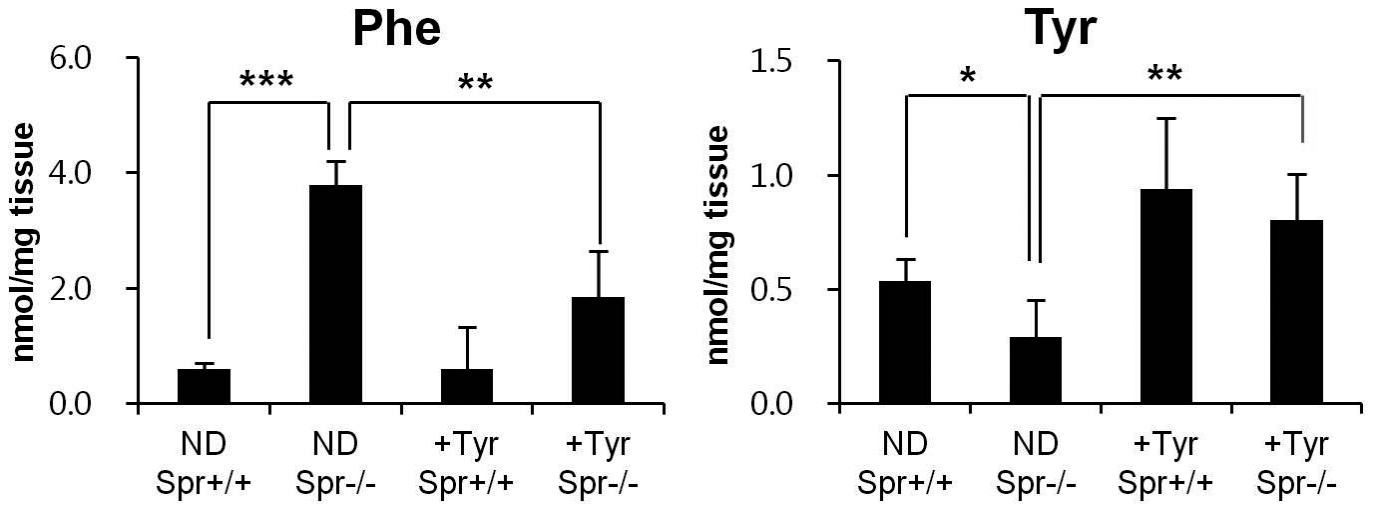
Restoration of brain tyrosine levels in BH_4_-deficient *Spr* ^−/−^ mice after the dietary tyrosine supplementation. Concentrations of phenylalanine and tyrosine in the cerebra separated from brains of *Spr* ^+/+^ or *Spr* ^−/−^ mice either fed a normal diet (ND) or the tyrosine diet (+Tyr) for 10 days are shown (n ≥5 for each experimental group). Data represent mean ± SD. Statistical significance was determined by Student’s *t* test, * P<0.05, ** P<0.01, *** P<0.001.

Since BH_4_ function is required for phenylalanine hydroxylase, which converts phenylalanine into tyrosine, it was anticipated that the availabilities of phenylalanine and tyrosine in brains of *Spr* ^−/−^ mice would be deranged compared to the wild type control *Spr* ^+/+^ mice. Phenylalanine levels in brains of *Spr* ^−/−^ mice fed a normal diet were remarkably higher than levels in brains of control *Spr* ^+/+^ mice, while tyrosine levels in brains of *Spr* ^−/−^ mice were much lower than those in the wild type *Spr* ^+/+^ counterparts. Brain tyrosine levels were increased in both *Spr* ^+/+^ and *Spr* ^−/−^ mice after the therapy relative to mice that did not receive tyrosine therapy. Contrarily, concentrations of brain phenylalanine were reduced in brains of *Spr* ^−/−^ mice given the dietary tyrosine therapy compared to *Spr* ^−/−^ mice fed a normal diet (Figure 1).

### Amelioration of Abnormal Motor Behaviors by the Tyrosine Therapy in BH_4_-deficient *Spr* ^−/−^ Mice

**Figure 2:**
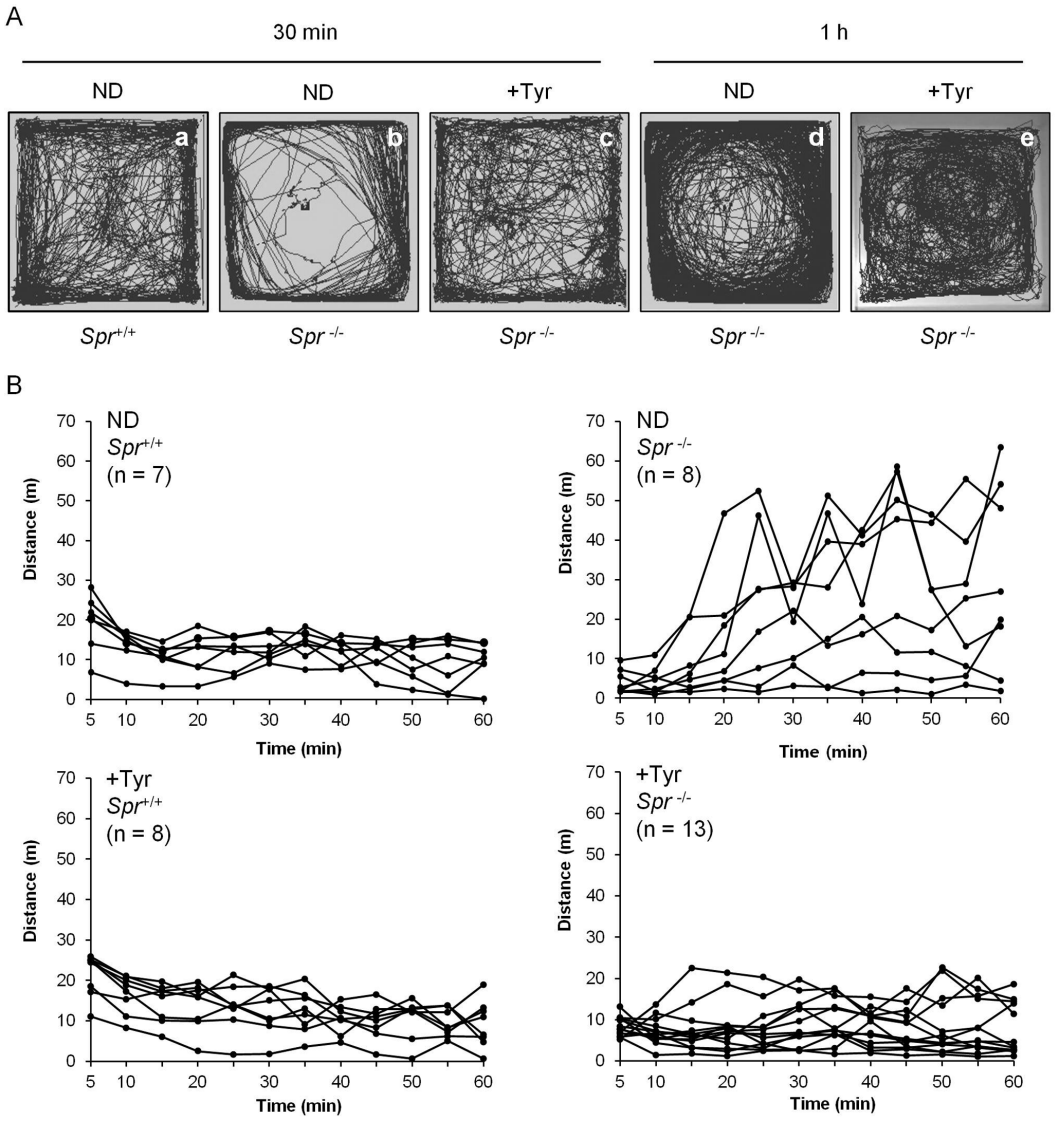
Abnormal open-field motor behaviors in *Spr* ^−/−^ mice were ameliorated by the dietary supplementation of tyrosine. Each experimental mouse was placed at the center of a square box (40×40×50 cm) made of white acryl in the dark. Each experimental group of mice was prepared as described in Materials and Methods. Newborn *Spr* ^−/−^ mice pretreated with ‘high dose of BH_4_’ for 25 days [19]. *Spr* ^+/+^ or *Spr* ^−/−^ mice 25 days of age were fed either a normal diet or the tyrosine diet for additional 10 days. A group of 35-d-old experimental mice fed either a normal diet (ND) or the tyrosine diet (+Tyr) was employed for the open-field test. (A) The representative locomotive patterns of wild-type control *Spr* ^+/+^ or mutant *Spr* ^−/−^ mice fed either a normal diet or the tyrosine diet are shown. The open-field test revealed a development of stereotypic circling behaviors in *Spr* ^−/−^ group of mice fed a normal diet. (B) Spontaneous movements during a time period of 30 min or 1 h were monitored by digital video recording. The distances traveled by experimental mice across the 5 min of observation period are shown.

**Figure 3:**
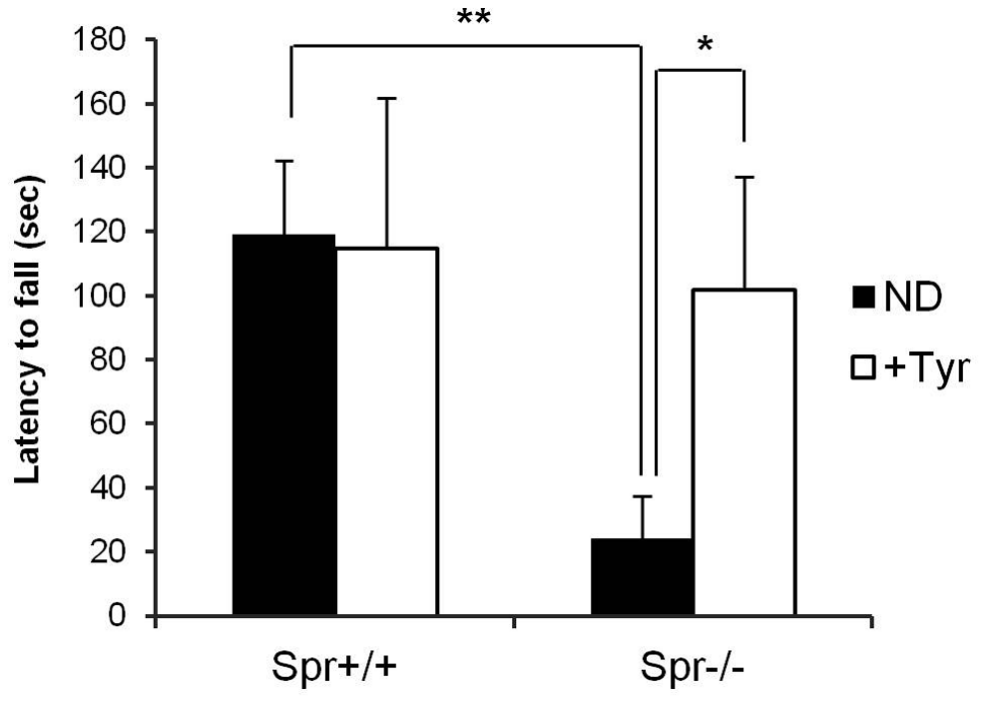
Improved rotating rod performance by dietary tyrosine therapy in *Spr* ^−/−^ mice. Mice were placed onto a rotating rod and the latency to fall was recorded. Each mouse performed 9 trials over a span of 3 days. Rotating rod performance by *Spr* ^+/+^ mice (n  = 5) or *Spr* ^−/−^ mice (n  = 5) fed a normal diet (ND) was compared with that by wild-type *Spr* ^+/+^ mice (n  = 5) or *Spr* ^−/−^ mice (n  = 6) that received the dietary tyrosine therapy (+Tyr). Data represent average time of latency to fall ± SD, * P<0.05, ** P<0.01.

**Figure 4:**
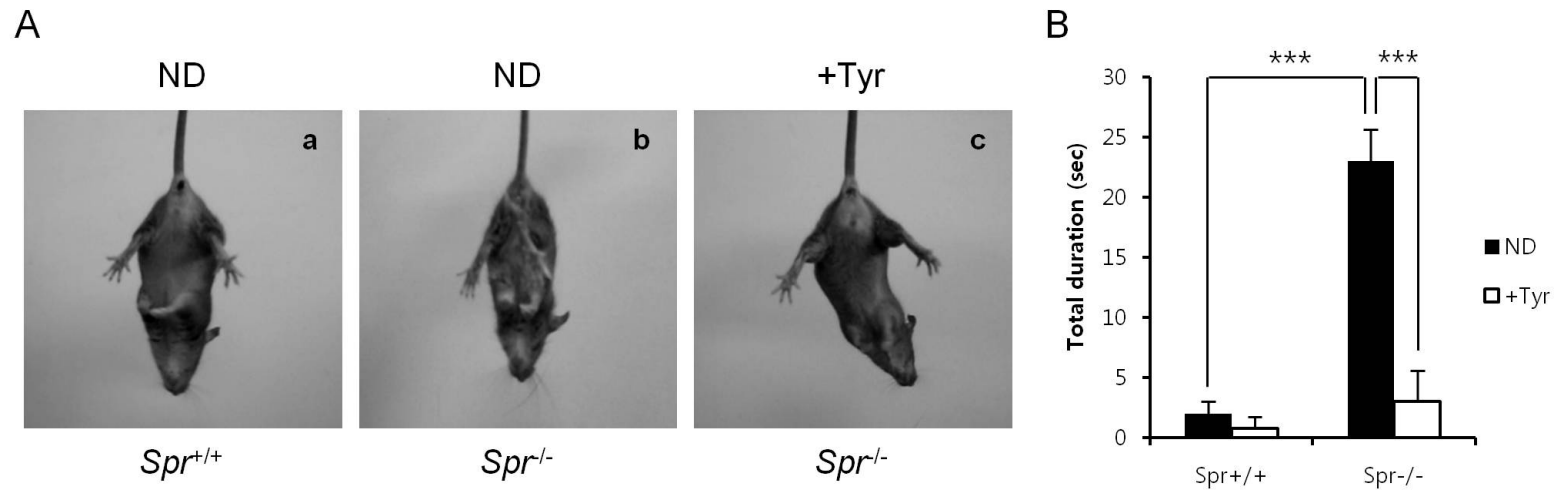
Hind-limb clasping test. Each experimental group of mice was suspended by the tail for 25 sec, and their abilities of hind-limb clasping were monitored by video recording. (A) Representative hind-limb clasping observed in *Spr* ^−/−^ mice fed a normal diet (ND) (b) but not in *Spr* ^+/+^ mice fed a normal diet (ND) (a) and *Spr* ^−/−^ mice fed the tyrosine diet (+Tyr) (c) is shown. (B) Total duration of hind-limb clasping during a 25 sec in *Spr* ^+/+^ mice fed a normal diet, *Spr* ^+/+^ mice fed the tyrosine diet and *Spr* ^−/−^ mice fed either a normal or the tyrosine diet (n  = 5 each) is shown. Values represent average time of duration ± SD, *** P<0.001.

We examined whether the motor impediments displayed by BH_4_-deficient mice whose brain tyrosine levels are insufficient can be improved by the dietary tyrosine supplementation. *Spr* ^−/−^ mice fed a normal diet displayed abnormal motor activities in the open-field during a 30 min or 1 h time period. Some *Spr* ^−/−^ mice fed a normal diet tended to avoid the center of the open-field, which is represented by their stereotypic circling behaviors (Figure 2A, b and d). The distance of spontaneous locomotive movements by experimental mice across the 5 min observation period in the open-field environment during the 1 h time period is shown in Figure 2B. The open-field test revealed that the dietary tyrosine supplementation did not affect the motor behaviors displayed by wild-type *Spr* ^+/+^ mice. However, locomotive movements by *Spr* ^−/−^ mice fed a normal diet varied among the mice. Some *Spr* ^−/−^ mice fed a normal diet displayed only basal levels of motor activities when compared to control *Spr* ^+/+^ mice fed a normal diet. Other *Spr* ^−/−^ mice fed a normal diet developed hyperactive movements in the open-field test (Figure 2B). Surprisingly, these controversial motor behaviors displayed by *Spr* ^−/−^ mice fed a normal diet were markedly normalized by the dietary supplementation of tyrosine for 10 days. Variance (S^2^) of total distance of locomotive movements tracked over a time period of 30 min or 1 h by *Spr* ^−/−^ mice fed a normal diet (S^2^ = 2535.4, S^2^ = 23625.6) became much smaller after the therapeutic tyrosine diet for 10 days (S^2^ = 586.9, S^2^ = 2831.5) (Table S1). In like manner, *Spr* ^−/−^ mice fed the therapeutic tyrosine diet performed comparably well on the rotating rod task after the dietary tyrosine therapy, compared with the performance by *Spr* ^−/−^ mice fed a normal diet (Figure 3). The rescue of motor dysfunction by the dietary tyrosine supplementation in *Spr* ^−/−^ mice was also shown by the hind-limb clasping test. Dystonic postures of hind-limbs with self-clasping were observed in most *Spr* ^−/−^ mice, whereas almost none of control *Spr* ^+/+^ mice exhibited hind-limb clasping phenotype. We observed that the hind-limb clasping phenotype in *Spr* ^−/−^ was substantially ameliorated after the dietary supplementation of tyrosine for 10 days (Figure 4).

### Brain Dopamine Levels were not Changed by the Dietary Tyrosine Therapy in *Spr* ^−/−^ Mice

**Figure 5:**
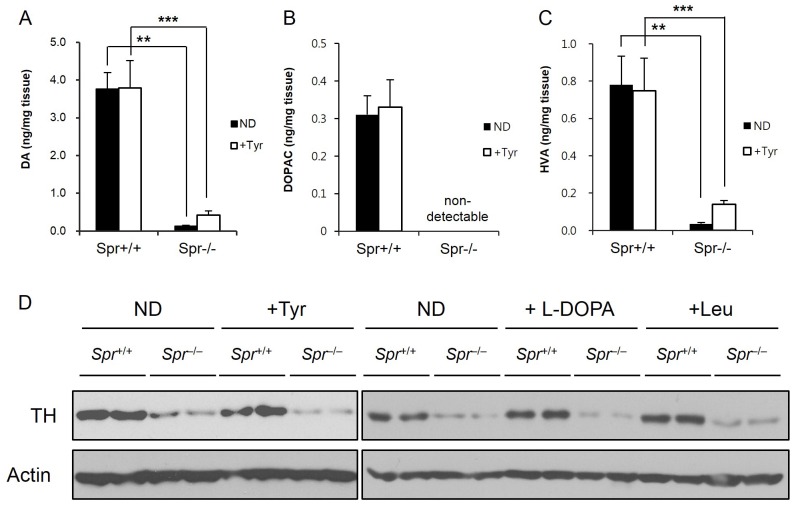
Levels of brain DA and its metabolites are irrelevant to the dietary tyrosine supplementation in *Spr* ^−/−^ mice. (A), (B), and (C) *Spr* ^+/+^ or *Spr* ^−/−^ mice were fed a normal (ND) or the tyrosine diet (+Tyr). Mice were analyzed for brain levels of DA (A), DOPAC (B), and HVA (C) in their caudate putamen (n ≥3). Values are means ± SD, ** P<0.01, *** P<0.001, compared with respective values in *Spr* ^+/+^ mice. (D) The brain levels of TH proteins in experimental mice were monitored by Western blot analysis. *Spr* ^+/+^ or *Spr* ^−/−^ mice were fed a normal diet or treated with the dietary tyrosine, L-DOPA, or leucine therapy for 10 days as described in Materials and Methods.

Dopamine (DA) is a classical neurotransmitter of the central nervous system and is essential for the body movement. We examined the hypothesis that the improved behavioral phenotypes in *Spr* ^−/−^ mice after the tyrosine diet are associated with the increase of brain dopamine levels. We measured levels of DA and its metabolites in the caudate putamen separated from brains of *Spr* ^+/+^ or *Spr* ^−/−^ mice fed either a normal or the therapeutic tyrosine diet. In accordance with the previous reports [4], [5], the levels of DA and its metabolites, 3,4-dihydroxyphenylacetic acid (DOPAC) and homovanillic acid (HVA) in the caudate putamen from brains of *Spr* ^−/−^ mice fed a normal diet were severely lower than the levels measured from the brains of control *Spr* ^+/+^ mice. In contrast to what we have expected, the levels of DA and its metabolites in brains of *Spr* ^−/−^ mice fed the therapeutic tyrosine diet were only slightly increased or above the detection limit. Despite the fact that the brain tyrosine levels in *Spr* ^−/−^ mice were restored after the dietary tyrosine therapy (Figure 1), the levels of brain DA and its metabolites were not substantially recovered in *Spr* ^−/−^ mice fed the tyrosine diet (Figure 5). In addition, the protein expression of tyrosine hydroxylase (TH), a rate-limiting enzyme for the production of DA [25], was investigated in the caudate putamen from experimental mice (Figure 5D). The amounts of TH protein were severely reduced in midbrains of *Spr* ^−/−^ mice fed either a normal or the tyrosine diet. The brain levels of TH in *Spr* ^−/−^ mice failed to recover after the tyrosine therapy. Neither the leucine diet nor the administration of dopamine precursor L-DOPA restored the diminished levels of TH protein in brains of *Spr* ^−/−^ mice.

### Enhanced Brain mTORC1 Activities in *Spr* ^−/−^ Mice by the Dietary Tyrosine Therapy

**Figure 6:**
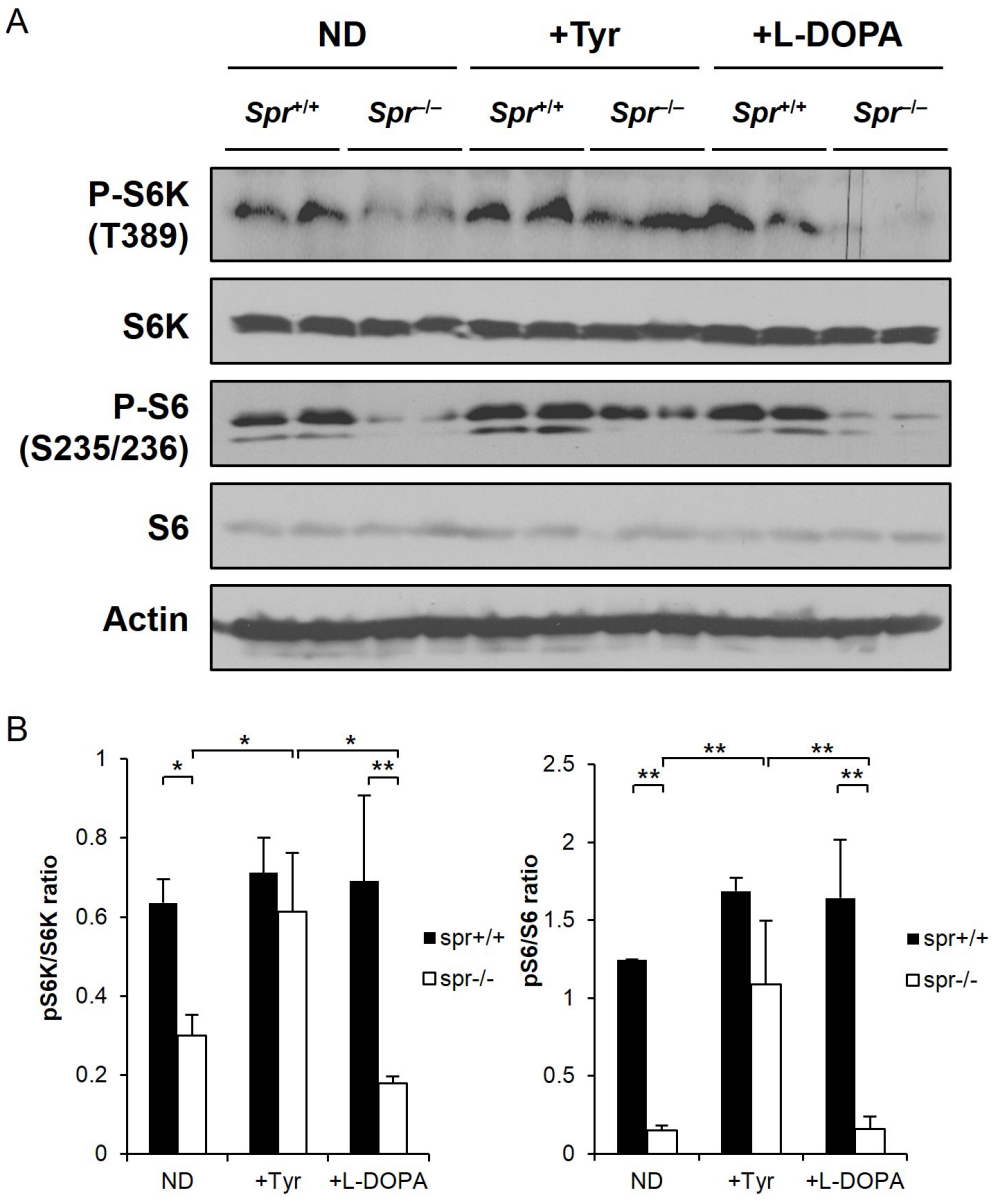
The dietary tyrosine supplementation enhances mTORC1 activity in brains of *Spr* ^−/−^ mice. (A) Immunoblotting was performed on the cerebral homogenates separated from brains of 35-d-old *Spr* ^+/+^ or *Spr* ^−/−^ mice. Each group of experimental mice was fed a normal diet (ND) or treated with the dietary tyrosine, or L-DOPA therapy for 10 days. Phosphorylation of S6 and S6K in the brain homogenates was examined to evaluate mTORC1 activity. The representative Western blot demonstrating mTORC1 recovery in *Spr* ^−/−^ mice by dietary tyrosine supplementation is shown. (B) The ratios of band intensities of pS6K/S6K and pS6/S6 were quantified by using image J software. Values are means ± SD (n ≥4 for each group of experimental mice). * P<0.05, ** P<0.01.

We explored whether the improved behavioral phenotypes in *Spr* ^−/−^ mice fed the therapeutic tyrosine diet correlate with brain mTORC1 activity. We compared mTORC1 activities in the cerebra from brains of *Spr* ^+/+^ or *Spr* ^−/−^ mice either fed a normal diet or the tyrosine diet by monitoring the phosphorylation of S6 and S6K. Phosphorylation of S6 at Ser235/236 and S6K at Thr389, which represent the activation of mTORC1 activity, was almost completely suppressed in brains of *Spr* ^−/−^ mice fed a normal diet. Interestingly, mTORC1 activities became activated in brains of *Spr* ^−/−^ mice after the tyrosine therapy for 10 days. Data also reveal that brain mTORC1 activities were not affected by the oral administration of L-DOPA in *Spr* ^−/−^ mice (Figure 6).

## Discussion

The present study indicates that the dietary supplementation of tyrosine ameliorates the abnormal motor behaviors exhibited by BH_4_-deficient mouse model. The cerebral concentrations of phenylalanine in *Spr* ^−/−^ mice were significantly higher than those in the wild-type *Spr* ^+/+^ control mice. Phenylalanine shares a common transporter to the brain with other LNAAs (large neutral amino acids) including tyrosine and also competes for the transport with other LNAAs across the blood brain barrier [26]. Amongst LNAAs, phenylalanine has the lowest Michaelis constant (Km) and is preferentially transported by the LNAA carrier protein [27]. Dietary supplementation of high-dose of tyrosine into *Spr* ^−/−^ mice not only elevated the cerebral levels of tyrosine but also lowered the cerebral phenylalanine levels (Figure 1).

Through a series of behavioral tests, we found that BH_4_-deficient *Spr* ^−/−^ mice display motor deficits such as variable and instable open-field behaviors (Figure 2), impaired motor coordination on rotating rod (Figure 3) and dystonic hind-limb clasping (Figure 4). The spontaneous locomotor activities in *Spr* ^−/−^ mice fed a normal diet in the open-field environment have provided conflicting results. These mice exhibited both hypo- and hyper-activity in the open-field test (Figure 2). Variance of total distance traveled in the open-field by *Spr* ^−/−^ mice was much greater than that by *Spr* ^+/+^ mice (Table S1). These complicated results seem to represent low baseline motor activity early during the initiation period of observation in *Spr* ^−/−^ mice as well as the impairment in the habituation to the test environment following the movement execution (Figure 2). The dietary effects on the amelioration of these motor deficits in BH_4_-deficient *Spr* ^−/−^ mice seem to be tyrosine-specific. We also examined the effects of the dietary leucine supplementation on the motor behaviors displayed by *Spr* ^−/−^ mice. The variable motor movements in the open-field and impaired motor coordination on the rotating rod displayed by *Spr* ^−/−^ mice were not improved by the dietary supplementation of leucine (Figure S1A–C).

Many behavioral disorders often are associated with abnormal neurotransmitter activity. For example, Parkinson’s disease is characterized by the degeneration of dopamine (DA) neurons in the brain [6]–[9]. Regulation of motor behaviors can be linked to a disruption of the nigrostriatal DA system and accounts for many motor impairments caused by the disruption of dopaminergic transmission [28], [29]. Bradykinesia or akinesia, the main neuropathological feature of Parkinson’s disease [30], and hyperactivity or hyperkinesia, a core feature of attention deficit hyperactivity disorder (ADHD) [31], are extremely oppositional phenotypes of abnormal motor behaviors. Interestingly, the neuropathological features of these two neurological disorders commonly include decreased DA activity in the brain [7], [30], [32]. We examined whether the improved motor activities by the dietary tyrosine supplementation in *Spr* ^−/−^ mice is linked with the increased brain DA levels in the experimental mice. The DA levels in the caudate putamen from brains of *Spr* ^−/−^ mice fed a normal diet were much lower than the levels in control *Spr* ^+/+^ mice. However, our data in Figure 5 do not support the hypothesis that the amelioration of abnormal motor behaviors in *Spr* ^−/−^ mice after the dietary tyrosine therapy stems from the recovery of brain DA concentrations since there was only modest increase in the DA levels in the caudate putamen from brains of *Spr* ^−/−^ mice after the dietary tyrosine supplementation. Our notion that amelioration of abnormal motor behavior is irrelevant to the brain DA levels in *Spr* ^−/−^ mice fed the tyrosine diet is strongly supported by independent experiments showing the dispensable roles of dietary supplementation of L-DOPA in open-field behaviors and rotating rod performances by *Spr* ^−/−^ mice (Figure S2). Since the brain TH protein and DA contents are known to be affected by BH_4_ deficiency, we performed Western blot analysis to examine whether the TH protein levels in the midbrains of *Spr* ^−/−^ mice were restored after the tyrosine therapy. Our data in Figure 5D indicate that brain TH protein levels in BH_4_-deficient *Spr* ^−/−^ mice are not affected by the dietary tyrosine treatment. The result further implies that the reduced levels of tyrosine conversion into L-DOPA and subsequent DA in *Spr* ^−/−^ mice were not influenced by tyrosine treatment (Figure 5A).

How the increased brain tyrosine levels in *Spr* ^−/−^ mice can biochemically correlate the improvement of abnormal motor behaviors in BH_4_-deficient mouse model is the least known aspect of this study. We have noticed that brain mTORC1 activities were pronouncedly increased in BH_4_-deficient *Spr* ^−/−^ mice by the dietary tyrosine treatment (Figure 6). Brain mTORC1 activity was restored neither by the leucine treatment (Figure S1D) nor by L-DOPA treatment (Figure 6) in these mutant mice suggesting tyrosine-dependent activation of brain mTORC1 activity in *Spr* ^−/−^ mice. The involvement of mTORC1 function in motor behaviors has been evidenced in some other mouse models. Both the deteriorative and beneficial effects of mTORC1 function on motor behaviors have been reported. Research by Santini et al. [33] provided evidence for the negative effects of mTORC1 signaling on motor function. They showed that the administration of rapamycin, an mTORC1 inhibitor, prevents the development of dyskinesia in a mouse model of Parkinsonism. The involvement of activated mTORC1 signaling in the behavioral abnormalities was further supported by the work showing the amelioration of behavioral abnormalities by rapamycin in *Pten*-knockout mice where the signals for cell growth, proliferation, and survival through phosphatidylinositol 3-kinase (PI3K) pathway were stimulated [34]. On the other hand, mTORC1 signaling has been implicated to be necessary for standardized motor behavior in mice. For example, S6K1-knockout mice generated by the knockout of the gene encoding mTORC1 substrate protein S6K1 have neurological and behavioral abnormalities [24]. Some human neurological disorders are also known to be associated with the dysregulation of mTORC1 signaling. Autistic disorders characterized by cognitive impairment and autism have been proposed to be the results of direct or indirect dysregulation of mTORC1 signaling [35]. Aberrant mTORC1 signaling observed in the postmorterm samples from brains of patients with Alzheimer’s disease, a neurodegenerative disorder characterized by gradual and severe loss of neurological function including memory and reasoning, implicates dysregulation of mTORC1 signaling as a biochemical feature of Alzheimer’s disease [36], [37]. Other studies have also postulated that human patients with neurological disorders display behavioral impairments and have dysregulated mTORC1 signaling [24], [34], [38], [39].

Amino acid availability appears to be necessary for the activation of mTORC1 and Rag GTPase, an amino acid-specific regulator of mTORC1 pathway [40], [41]. Since mTORC1 signaling is regarded as the most important regulator of protein synthesis and its degradation, dysregulation of mTORC1 signaling may result in significant synaptic plasticity, autophagy and ubiquitin-mediated proteolysis in the brain [42]. Thus, it is a reasonable expectation that the availability of brain amino acids, which signal mTORC1 pathway in the brain may play an important role in the behavioral deficits associated with many neurodegenerative diseases.

## Supporting Information

Figure S1 **Dietary effect of leucine on motor behaviors displayed by** ***Spr*** **^−/−^ mice.** For dietary leucine supplementation, *Spr* ^+/+^ or *Spr* ^−/−^ mice 25 days of age were fed the leucine supplemented diet in which 3.6% leucine (w/w) was added to normal diet for 10 days under *ad libitum* conditions. (A, B) Abnormal open-field behaviors in *Spr* ^−/−^ mice were not improved by the dietary supplementation of leucine. The experimental details were the same as in Figure 2. (C) Dietary supplementation of leucine fails to improve rotating rod performance displayed by *Spr* ^−/−^ mice. The experimental details were the same as in Figure 3. ***P<0.001. (D) Dietary leucine supplementation has no effect on brain mTORC1 activity in *Spr* ^−/−^ mice. Brain mTORC1 activities were measured using the phosphorylation of S6 as a readout. Other experimental procedures were the same as in Figure 6.
(TIF)

Figure S2 **Dietary effect of L-DOPA on motor behaviors by** ***Spr*** **^−/−^ mice.** Both strains of *Spr* ^+/+^ and *Spr* ^−/−^ mice 25 days of age were orally administrated L-DOPA (13.5 µg/g body weight/day) for 10 days. Open-field behaviors (A) or rotating rod performance (B) displayed by the experimental mice (n  = 2 for each experimental group) are shown. Experimental conditions were the same as in Figure 2 and Figure 3. ***P<0.001.
(TIF)

Table S1 **Hypervariable distances moved by** ***Spr*** **^−/−^ mice in the open-field test become normalized after the tyrosine therapy.**
(DOCX)
