# Supplementary figures and images for: Amelioration of Behavioral Abnormalities in BH4-deficient Mice by Dietary Supplementation of Tyrosine
Source: PLoS One. 2013 Apr 5;8(4):e60803. doi: 10.1371/journal.pone.0060803 (PMC3618182; doi:10.1371/journal.pone.0060803)

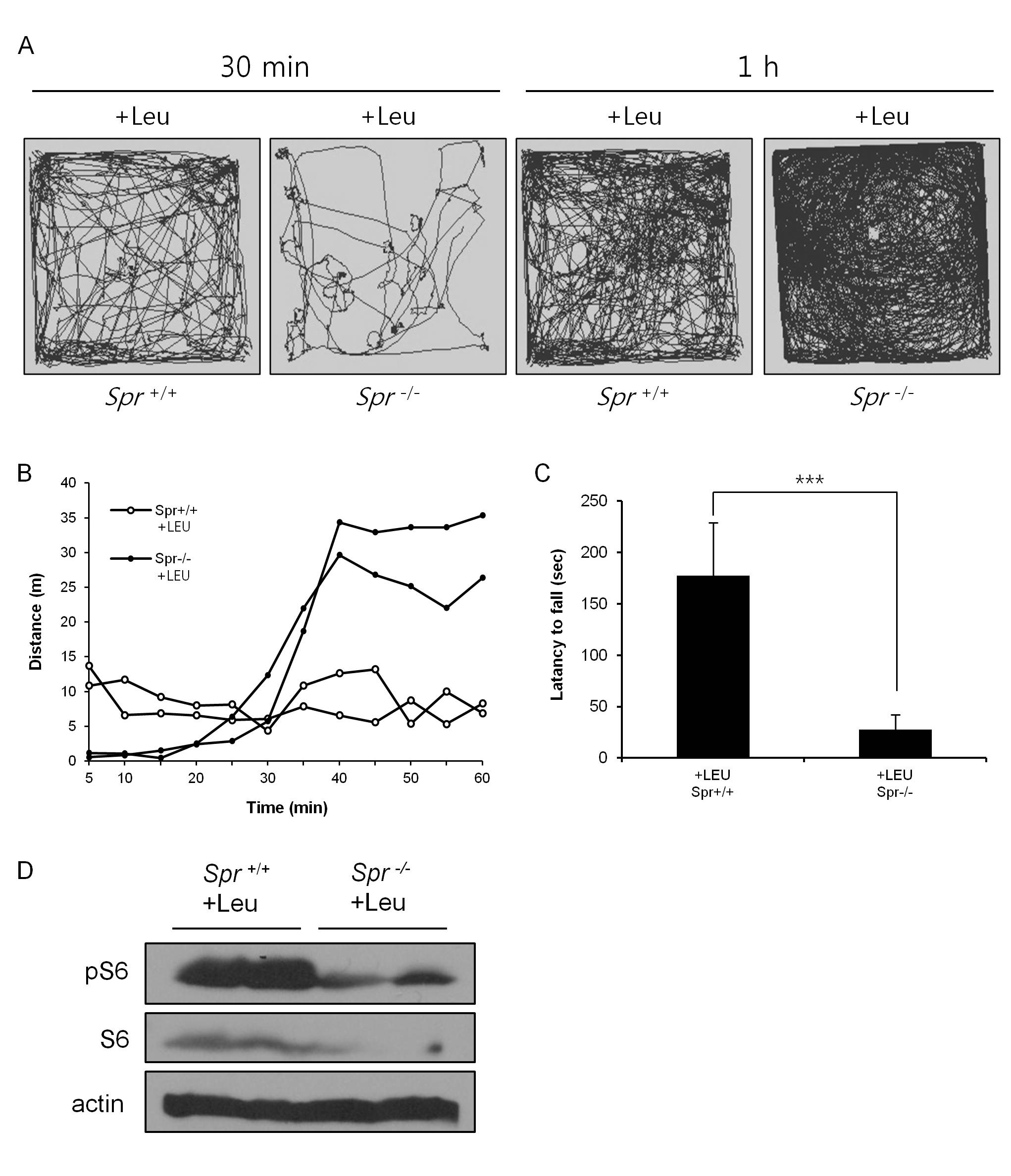

Supplement: Figure S1 — Dietary effect of leucine on motor behaviors displayed by Spr −/− mice. For dietary leucine supplementation, Spr +/+ or Spr −/− mice 25 days of age were fed the leucine supplemented diet in which 3.6% leucine (w/w) was added to normal diet for 10 days under ad libitum conditions. (A, B) Abnormal open-field behaviors in Spr −/− mice were not improved by the dietary supplementation of leucine. The experimental details were the same as in Figure 2. (C) Dietary supplementation of leucine fails to improve rotating rod performance displayed by Spr −/− mice. The experimental details were the same as in Figure 3. ***P<0.001. (D) Dietary leucine supplementation has no effect on brain mTORC1 activity in Spr −/− mice. Brain mTORC1 activities were measured using the phosphorylation of S6 as a readout. Other experimental procedures were the same as in Figure 6. (TIF) [file pone.0060803.s001.tif]

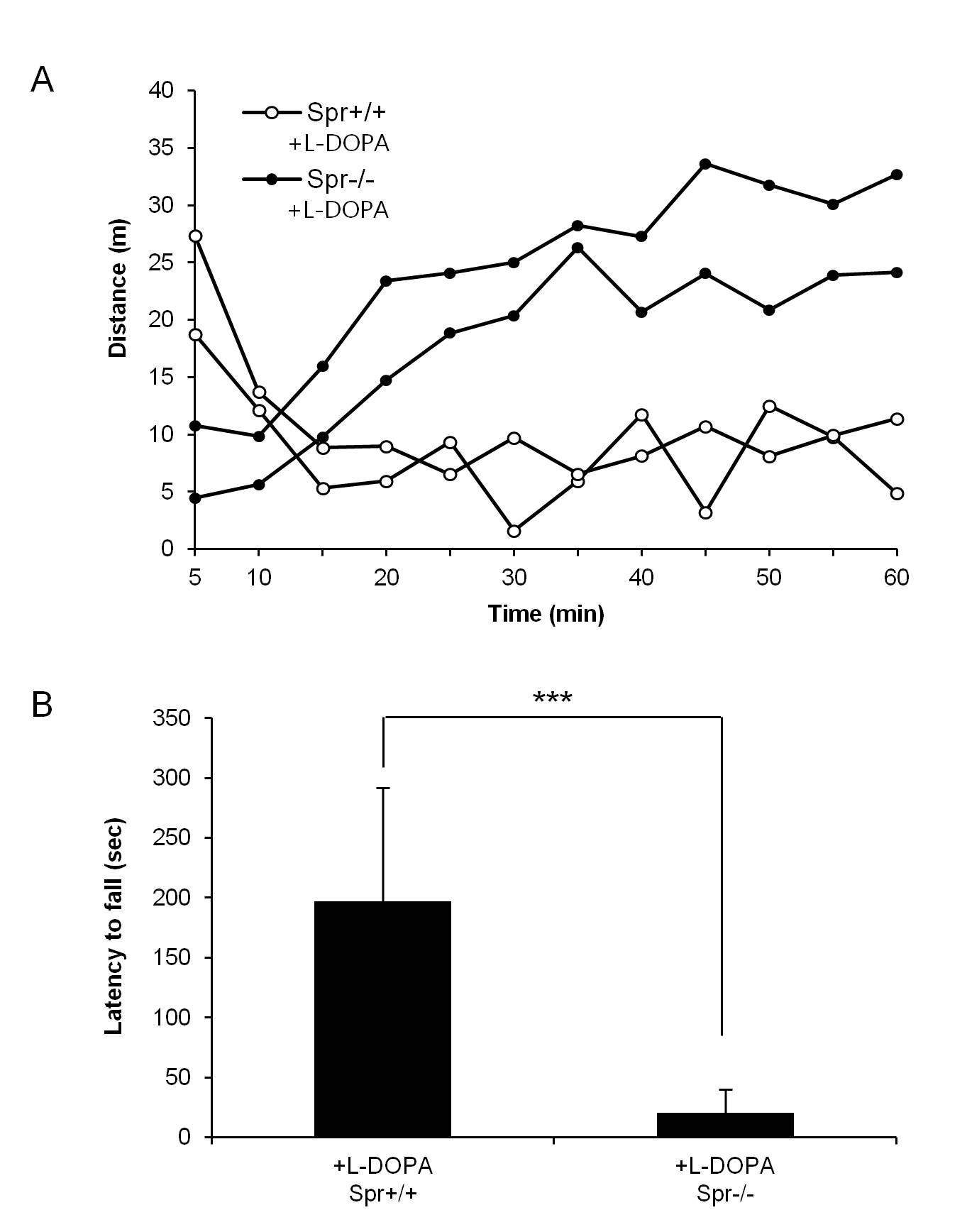

Supplement: Figure S2 — Dietary effect of L-DOPA on motor behaviors by Spr −/− mice. Both strains of Spr +/+ and Spr −/− mice 25 days of age were orally administrated L-DOPA (13.5 µg/g body weight/day) for 10 days. Open-field behaviors (A) or rotating rod performance (B) displayed by the experimental mice (n = 2 for each experimental group) are shown. Experimental conditions were the same as in Figure 2 and Figure 3. ***P<0.001. (TIF) [file pone.0060803.s002.tif]
