# Supplementary material for: Amelioration of Behavioral Abnormalities in BH4-deficient Mice by Dietary Supplementation of Tyrosine
Source: PLoS One. 2013 Apr 5;8(4):e60803. doi: 10.1371/journal.pone.0060803 (PMC3618182; doi:10.1371/journal.pone.0060803)
Supplement: Table S1 — Hypervariable distances moved by Spr −/− mice in the open-field test become normalized after the tyrosine therapy. (DOCX) [file pone.0060803.s003.docx]

Table S1. Hypervariable distances moved by *Spr*^-/-^ mice in the open-field test become normalized after the tyrosine therapy.

| Genotype | *Spr*^+/+^ (n=7) | *Spr*^+/+^ (n=8) | *Spr*^-/-^ (n=8) | *Spr*^-/-^ (n=13) |
| --- | --- | --- | --- | --- |
| Diet | ND | +Tyr | ND | +Tyr |
| Average distance moved for 30 min (m) | 79.4±24.2 | 95±29.9 | 72.5±50.8 | 47.9±24.2 |
| Distance Variance (S^2^) | 583.8 | 896.7 | 2535.4 | 586.9 |
|  |  |  |  |  |
| Average distance moved for 1 h (m) | 145.4±42.8 | 164.5±58.3 | 227.4±153.7 | 97.4±53.2 |
| Distance Variance (S^2^) | 1832.2 | 3396.9 | 23625.6 | 2831.5 |

Motor movements in the open-field test in the dark were tracked over for a time period of 30 min or 1 h. It should be noted that the Variance (S^2^) of locomotive movements displayed by *Spr*^-/-^ mice fed a normal diet are much greater than those by *Spr*^-/-^ mice fed the therapeutic tyrosine diet.
